# Asymmetric Dual-Band Tracking Technique for Optimal Joint Processing of BDS B1I and B1C Signals

**DOI:** 10.3390/s17102360

**Published:** 2017-10-16

**Authors:** Chuhan Wang, Xiaowei Cui, Tianyi Ma, Sihao Zhao, Mingquan Lu

**Affiliations:** Department of Electronic Engineering, Tsinghua University, Beijing 100084, China; wangch13@mails.tsinghua.edu.cn (C.W.); matianyi@mail.tsinghua.edu.cn (T.M.); zsh_thu@mail.tsinghua.edu.cn (S.Z.); lumq@mail.tsinghua.edu.cn (M.L.)

**Keywords:** BeiDou Navigation Satellite System, ASYM Dual-Band Tracking, tracking channel architecture, Gabor Bandwidth

## Abstract

Along with the rapid development of the Global Navigation Satellite System (GNSS), satellite navigation signals have become more diversified, complex, and agile in adapting to increasing market demands. Various techniques have been developed for processing multiple navigation signals to achieve better performance in terms of accuracy, sensitivity, and robustness. This paper focuses on a technique for processing two signals with separate but adjacent center frequencies, such as B1I and B1C signals in the BeiDou global system. The two signals may differ in modulation scheme, power, and initial phase relation and can be processed independently by user receivers; however, the propagation delays of the two signals from a satellite are nearly identical as they are modulated on adjacent frequencies, share the same reference clock, and undergo nearly identical propagation paths to the receiver, resulting in strong coherence between the two signals. Joint processing of these signals can achieve optimal measurement performance due to the increased Gabor bandwidth and power. In this paper, we propose a universal scheme of asymmetric dual-band tracking (ASYM-DBT) to take advantage of the strong coherence, the increased Gabor bandwidth, and power of the two signals in achieving much-reduced thermal noise and more accurate ranging results when compared with the traditional single-band algorithm.

## 1. Introduction

It is well known that the widespread application of the Global Navigation Satellite System (GNSS) has promoted the construction, improvement, and modernization of satellite navigation systems such as GPS and Galileo in major countries and regions of the world [1,2]. Among them, China’s BeiDou system has seen rapid development in recent years. The BeiDou regional system began its service to the Asia Pacific region in 2012, while the BeiDou global system has entered the deployment phase in 2017 and will complete global networking in 2020 [3,4]. On the premise of guaranteed forward compatibility of open service to legacy users, the BeiDou global system will broadcast several new navigation signals in the B1, B2, and B3 frequency bands to achieve better performance in terms of accuracy, sensitivity, and robustness [5]. Of particular note is that two civilian navigation signals—B1I and B1C—modulated at different center frequencies in the B1 frequency band will be provided in the BeiDou global system. While the B1I signal [6], with a center frequency of 1561.098 MHz, is retained from the regional system to offer service to legacy users, the new B1C signal, with a center frequency of 1575.42 MHz and which is implemented by the state-of-the-art multiplexed binary offset carrier (MBOC) modulation scheme, will be broadcasted mainly to offer interoperability with GPS L1C and Galileo E1C signals [7].

Traditionally, each satellite in the GNSS constellation broadcasts multiple navigation signals in the same or different frequency bands. For example, GPS broadcasts both a L1C/A signal and a L1P (Y) signal in the L1 band while broadcasting a L2P (Y) signal in the L2 band; in the modernization of GPS, the newly added L5 signal contains both data and pilot channels, which can also be viewed as a special case of broadcasting two signals at the same frequency [8]. Because two or more navigation signals broadcast by one satellite are generated based on the same onboard reference clock and their propagation paths to the receiver antenna are nearly the same, the time delay and Doppler-shift of these signals at the receiver are strongly correlated. This is the basis for the joint processing of multiple signals to achieve better measurement performance.

Various techniques have been developed for the joint processing of multiple navigation signals, and they can be grouped into three types. The first type of joint processing is based on a tracking loop. The time delay and Doppler-shift parameters obtained by tracking a strong signal are used to assist in tracking the weak signal. One typical example is the GPS receiver with dual-frequency positioning, which utilizes the L1 signal to assist semi-codeless tracking of the L2P(Y) signal [8,9,10]. This type of joint processing handles two signals separately in essence, and can improve tracking sensitivity for the weak signal at the parameter level. The second type of joint processing is based on code and/or phase discriminators. For instance, when receiving the L5 signal, the outputs from code and phase discriminators of the data and pilot channels can be coherently or non-coherently combined to improve the accuracy and sensitivity of tracking [11,12]. The third type is based on the auto-correlation function (ACF). By quasi-coherently combining correlator outputs of L1C and L1C/A signal components, the proposed algorithm in [13] successfully constructs a synthesis correlation function with no side-peaks. In addition, it combines the power of two signals to improve the tracking accuracy. These three types of joint processing implement different ways of jointly utilizing the power of multiple signals. We hence refer to this kind of performance improvement as power gain.

The unique features of the B1I and B1C signals in the BeiDou global system, however, offer more potential than can be exploited by the three schemes described above. First, compared to GPS L5 data/pilot channels or L1C/A and L1C signals, B1I and B1C signals are modulated at different center frequencies in the B1 frequency band [6,14], which brings about a bandwidth gain in addition to power gain. In addition, the center frequencies of B1I and B1C signals are separated by only 14.322 MHz, which is much closer than the ≥300 MHz separation between the center frequencies of the GPS L1C/A and L2P(Y) signals. As a result, both signals can be received by one broadband radio channel simultaneously, and the ionosphere delays experienced by these two signals are almost identical. To take full advantage of such unique characteristics of B1I and B1C, a better joint processing approach is clearly desired.

In this paper we first propose to view B1I and B1C signals as one virtual navigation signal with Asymmetric Dual Sidebands, named B1-ADS. The theoretical analysis in the following shows that the Gabor bandwidth of this virtual asymmetric signal is much wider than those of B1I and B1C, and even wider than those of signals modulated by Binary Phase Shift Keying (BPSK) (10) such as L1P(Y) and L5. This indicates that the ranging accuracy of this virtual signal is much higher than that of B1I or B1C alone. What needs to be emphasized here is that the performance improvement is mainly due to bandwidth gain rather than power gain. Although the impressive potential for improved ranging accuracy is indicated by the result of theoretical analysis, it still remains a big challenge in current receiver design to implement optimal reception processing for B1-ADS signals. On one hand, the traditional joint processing methods mentioned above, such as tracking loop parameter aiding or combining discriminator outputs of different signals, fail to take advantage of the bandwidth gain for the B1-ADS signal. On the other hand, although this B1-ADS signal has the frequency separation characteristic similar to the BOC-modulated signal, the complexity of its ACF caused by the asymmetry between the upper and lower sidebands renders those classical reception techniques of the BOC signal, such as Bump-Jump [15] and the Double Estimator Technique (DET) [16], impossible to apply directly.

So, in this paper, we further present a novel ASYMmetric Dual-Band Tracking technique to achieve the optimal reception processing of B1-ADS signals through harvesting both power gain and bandwidth gain, which is abbreviated as ASYM-DBT. The key of ASYM-DBT is to construct discriminators of code delay, subcarrier, and carrier by coherently combining the correlator outputs of dual sideband signals and to track these three components by delay lock loop (DLL), subcarrier lock loop (SLL) and phase lock loop (PLL), respectively. The much-improved ranging accuracy committed by the B1-ADS signal is obtained by combining the outputs of the code delay and the subcarrier tracking loops. In fact, ASYM-DBT is an extension of our team’s previously proposed Dual BPSK Tracking method (DBT) [17] designed for optimal reception of an alternative BOC (AltBOC) signal. The DBT method adopts the ideas of DET and double phase estimator (DPE) [18] to treat the subcarrier of BOC signals independently and to track it by an additional loop, but the characteristic of no need to generate a subcarrier makes this method have lower hardware complexity and more generality. A similar method to DBT is also mentioned in [19]. As an extension of the original DBT method, ASYM-DBT has been improved to cope with the more complicated asymmetric case of dual-sideband signals with different modulation schemes, power, and initial phase relations.

The remainder of this paper is organized as follows: in Section 2, the BDS B1I and B1C signal properties are described and the power spectral density (PSD), ACF, and Gabor bandwidth of the whole B1-ADS signal are given and analyzed. In Section 3, we propose a reception model for the B1-ADS signal. In Section 4, the ASYM-DBT method is described in detail, including tracking channel architecture and its theoretical thermal noise performance. In Section 5, a software-defined receiver developed by Tsinghua University is used to process B1I and B1C signals from a signal generator. A simulated experiment is designed to verify the theoretical thermal noise performance of the ASYM-DBT method. Finally, conclusions are drawn in Section 6.

## 2. Summary of the BDS B1 Signal 

As mentioned above, two civilian signals, B1I and B1C, will be broadcast simultaneously in the B1 frequency band in the future BeiDou global system [14]. From the viewpoint of the designer of the BeiDou system, the B1I and B1C signals have been designed independently and are expected to be processed in receivers as two separate signals. In this section, the characteristics of the B1I and B1C signals will be introduced respectively, and then the characteristics of the virtual broadband signal B1-ADS composed of B1I and B1C will be analyzed and its excellent potential for ranging performance will be discussed.

### 2.1. Brief Introduction of the B1I and B1C Signals

The basic characteristics of the B1I and B1C signals are given in Table 1.

The B1I signal has been a major civilian signal of the BeiDou regional system and will be retained in the BeiDou global system for legacy users. The B1I signal adopts the classical BPSK modulation with a chip rate $f_{code}$ of 2.046 MHz, usually written as BPSK(2). The B1I ranging code has 2046 chips and is a balanced Gold code truncated with the last chip. The center frequency of the B1I signal is 1561.098 MHz [20,21]. The details of the B1I signal can be found in the official documentation [6]. The PSD of B1I with BPSK(2) modulation can be expressed as follows [22]:

(1) $$G_{B1I}(f)=f_{code}\frac{\sin^{2}(\frac{\pi f}{f_{code}})}{{\left(\pi f\right)}^{2}}$$

The new B1C signal, modulated on the carrier with a center frequency of 1575.42 MHz, will be broadcast in the BeiDou global system. The B1C signal incorporates the innovative data-plus-pilot signal structure, and contains 25% power in the data component and 75% in the pilot component. Both signal components are found to repeat after 10,230 chips and spread at the code chip rate $f_{code}$ of 1.023 Mcps. While the data component is generated with the BOC(1,1) modulation with a 1.023 Mcps square wave subcarrier frequency, the pilot component modulation, called multiplexed BOC (MBOC) [7] has a spectrum produced by a BOC(1,1) component and a BOC(6,1) component with a 6.138 Mcps square wave subcarrier frequency. Time-multiplexed BOC (TMBOC) is used in the new-generation BeiDou-3 experimental satellites, and is produced by replacing four of the 33 spreading symbols with BOC(6,1) modulation while retaining BOC(1,1) modulation for all other spreading symbols [5,14]. However, in the future BeiDou global system, QMBOC with BOC(1,1) and BOC(6,1) modulated on the orthogonal phase of the carrier will be adopted [23]. The PSD of B1C, with 1/11 of the total power in BOC(6,1) and the remaining 10/11 of the total power in BOC(1,1), can be expressed as follows [22]:

(2) $$\begin{array}{ll} G_{B1C}(f) & =\frac{10}{11}G_{BOC(1,1)}(f)+\frac{1}{11}G_{BOC(6,1)}(f)\\ & =\frac{f_{code}}{11\pi^{2}f^{2}}\sin^{2}(\frac{\pi f}{f_{code}})\left[10\tan^{2}\left(\frac{\pi f}{2f_{code}}\right)+\tan^{2}\left(\frac{\pi f}{12f_{code}}\right)\right] \end{array}$$

### 2.2. Characteristic Analysis of the Virtual Broadband Signal B1-ADS

Admittedly, B1I and B1C signals with unequal power and different modulation schemes have been expected to be processed as two independent signals in receivers. However, when considering the fact that both signals are generated based on the same onboard reference clock and their propagation paths to the receiver antenna are nearly the same, we can regard these two signals as a whole and define it as a new signal, that is, B1-ADS. This virtual wideband signal has a center frequency of 1568.259 MHz ((1561.098 + 1575.42)/2) and has two asymmetrical sidebands. The characteristics of the B1-ADS signal are given in terms of PSD, Gabor bandwidth, and ACF, as follows.

**Figure 1:**
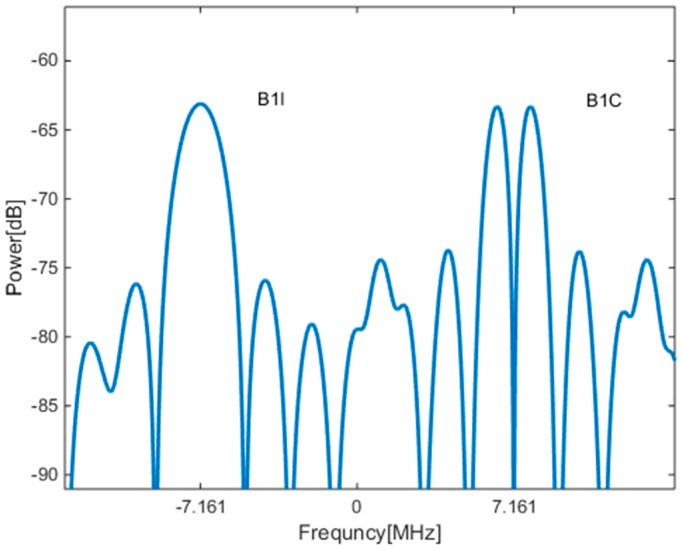
The power spectral density (PSD) of the B1-ADS signal.

First, the PSD of the B1-ADS signal is approximated by the sum of the PSDs of the B1I and B1C signals, as shown below:

(3) $$G_{B1-ADS}(f)=G_{B1I}(f+f_{s})+G_{B1C}(f-f_{s})$$

where $f_{s}$ represents half the center frequency spacing between the B1I and B1C signals, i.e., 7.161 MHz. The rationality of this approximation is that the center frequencies of the B1I and B1C signals are spaced by 14.332 MHz, and the vast majority of the spectrum of the B1I and B1C signals does not overlap, which leads to negligible interaction between the two components of the spectrum. The PSD of the B1-ADS signal is plotted in Figure 1 where it can be seen that the two components, B1I and B1C, are modulated on the lower and upper sidebands, respectively.

Then, the Gabor bandwidth, as an important index used to characterize a signal’s theoretical ranging capability, can be calculated based on its PSD. It is well known that the broader the Gabor bandwidth is, the better the ranging performance that can be achieved. Given the PSD and front-end bandwidth $\beta_{r}$, the formula to calculate Gabor bandwidth is as follows [24,25]:

(4) $${\bar{F}}^{2}=\frac{\int_{-\beta_{r}/2}^{+\beta_{r}/2}f^{2}G(f)df}{\int_{-\beta_{r}/2}^{+\beta_{r}/2}G(f)df}.$$

**Figure 2:**
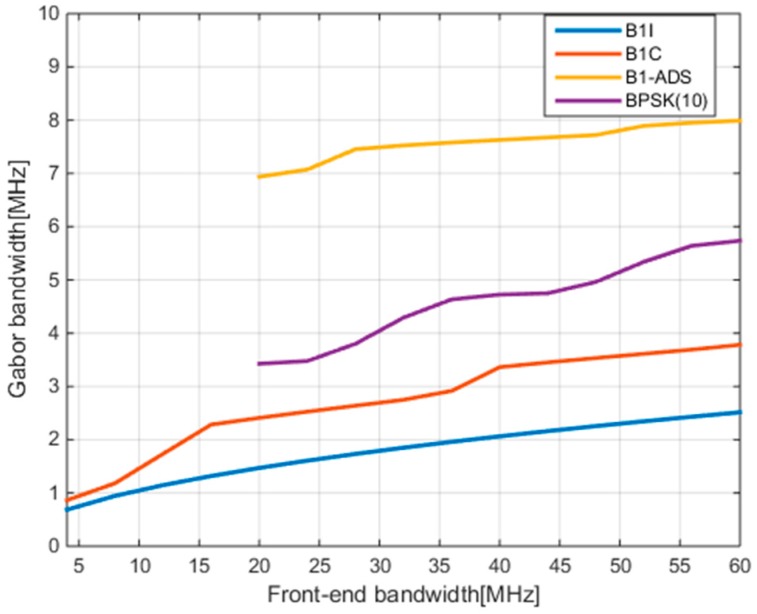
The Gabor bandwidths of B1-ADS and other signals.

Figure 2 gives the results of the Gabor bandwidth of the B1-ADS signal with different front-end bandwidths. At the same time, the Gabor bandwidth results of B1I, B1C, and BPSK(10) modulation signal are also given in the same figure for comparison. Among them, the Gabor bandwidths of two wideband signals, B1-ADS and BPSK(10), are calculated for the front-end bandwidths in the [20–60] MHz range. Figure 2 shows that the Gabor bandwidth of B1-ADS is not only significantly greater than those of the B1I and B1C signals as expected, but also greater than the Gabor bandwidth of the conventional wideband signal with BPSK(10) modulation. This indicates the excellent potential for ranging performance of the B1-ADS signal.

**Figure 3:**
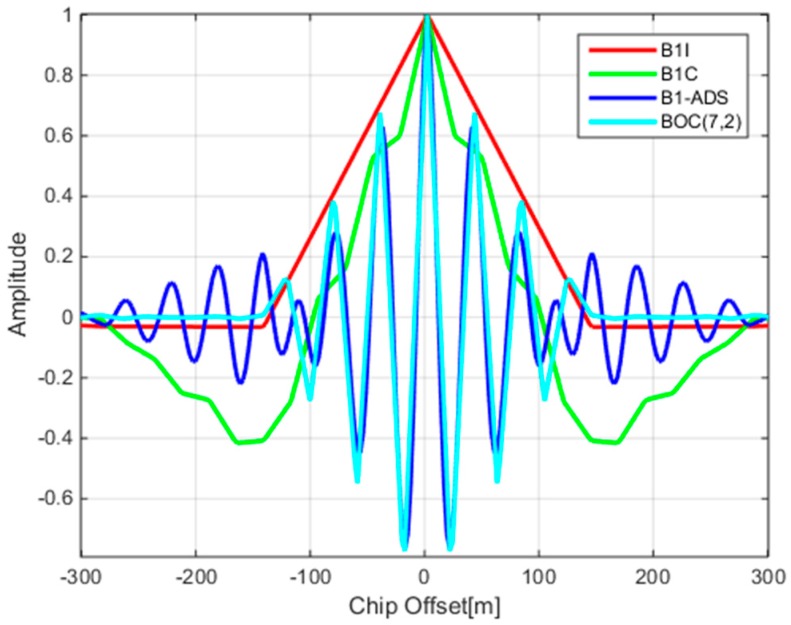
The auto-correlation functions (ACFs) of B1-ADS and other signals.

Finally, the ACF of the B1-ADS signal is depicted in Figure 3. The same figure also gives the ACFs of B1I, B1C, and a signal with BOC(7,2) modulation. Comparing the ACFs of these signals, it is not difficult to see that the ACF of the B1-ADS has multiple peaks similar to that of the BOC(7,2) signal. The reason will be explained in the next section. While the steep main peak in the ACF of B1-ADS signal is another indicator of its excellent ranging performance, the feature of multiple peaks can also lead to the problem of tracking ambiguity similar to BOC signals.

## 3. Reception Model of B1-ADS Signal

While the theoretical analysis of the frequency domain and ACF in the previous section shows that the B1-ADS signal has an excellent potential for ranging accuracy, we will further discuss the time domain reception model of the B1-ADS signal in this section for designing the optimal reception algorithm. As mentioned earlier, the B1C signal adopts the data-plus-pilot signal structure, and the pilot signal also includes the components of BOC(1,1) and BOC(6,1). To facilitate subsequent analysis and expression, without losing generality, a pilot tracking and data demodulation strategy that neglects the BOC(6,1) component will be adopted for the B1C signal. In other words, the B1-ADS signal discussed below will be simplified to consisting of the B1I signal on the lower band and the BOC(1,1) component of the B1C pilot signal on the upper band.

In this paper, the B1-ADS signal is assumed to be received in the presence of thermal noise at the receiver front end that can be modeled as stationary additive white Gaussian noise. As a result, the received signal can be expressed as follows:

(5) $$r\left(t\right)=s_{B1-ADS}\left(t-\tau \right)+n\left(t\right)$$

where τ is the signal propagation delay, and n(t) is the thermal noise. In Equation (6), the delayed B1-ADS signal is represented as a composite of B1I and B1C signals:

(6) $$\begin{array}{cc} s_{B1-ADS}\left(t-\tau \right)= & A_{B1I}s_{B1I}\left(t-\tau \right)\cos\left(2\pi f_{B1I}\left(t-\tau \right)+\theta_{B1I}\right)\\ & +A_{B1C}s_{B1C}\left(t-\tau \right)\cos\left(2\pi f_{B1C}\left(t-\tau \right)+\theta_{B1C}\right) \end{array}$$

where f, θ and A represent received frequency, initial carrier phase, and amplitude of the corresponding components, respectively. The values $s_{B1I}\left(t-\tau \right)$ and $s_{B1C}\left(t-\tau \right)$ are the baseband waveforms of the B1I and B1C signals, which are the combination of subcarrier, ranging code, secondary code, and navigation data bit. The received frequency may be further expressed as a sum of the transmitted nominal frequency $f_{\cdot ,n}$ and Doppler frequency $f_{\cdot ,D}$ as follows,

(7) $$\left\{ \begin{array}{l} f_{B1I}=f_{B1I,n}+f_{B1I,D}\\ f_{B1C}=f_{B1C,n}+f_{B1C,D} \end{array}\right.$$

Next, we want to rewrite Equation (6) with the parameters of the B1-ADS signal. The nominal carrier frequency and subcarrier frequency of the B1-ADS signal are introduced and defined in Equation (8), and the initial phases of carrier and subcarrier are given in Equation (9):

(8) $$\left\{ \begin{array}{l} f_{c,n}=\frac{f_{B1C,n}+f_{B1I,n}}{2}=1568.259\mathrm{MHz}\\ f_{s,n}=\frac{f_{B1C,n}-f_{B1I,n}}{2}=7.161\mathrm{MHz} \end{array}\right.$$

(9) $$\left\{ \begin{array}{l} \theta_{c}=\frac{\theta_{B1C}+\theta_{B1I}}{2}\\ \theta_{s}=\frac{\theta_{B1C}-\theta_{B1I}}{2} \end{array}\right.$$

where the subscripts c and s are used to indicate carrier and subcarrier, respectively. Since the Doppler frequency scales linearly with the signal center frequency, the Doppler frequencies of both the carrier and subcarrier of B1-ADS signal can be represented in terms of the Doppler frequencies of the B1I and B1C signals as follows:

(10) $$\left\{ \begin{array}{l} f_{c,D}=\frac{f_{B1C,D}+f_{B1I,D}}{2}\\ f_{s,D}=\frac{f_{B1C,D}-f_{B1I,D}}{2} \end{array}\right.$$

Then, the received frequencies of the carrier and subcarrier of the B1-ADS signal are given in Equation (11),

(11) $$\left\{ \begin{array}{l} f_{c}=f_{c,n}+f_{c,D}\\ f_{s}=f_{s,n}+f_{s,D} \end{array}\right.$$

By Equations (7)–(11), Equation (6) is rewritten based on the parameters of the B1-ADS signal as:

(12) $$\begin{array}{cc} s_{B1-ADS}\left(t-\tau \right)= & A_{B1I}s_{B1I}\left(t-\tau \right)\cos\left(2\pi \left(f_{c}-f_{s}\right)\left(t-\tau \right)+\theta_{c}-\theta_{s}\right)\\ & +A_{B1C}s_{B1C}\left(t-\tau \right)\cos\left(2\pi \left(f_{c}+f_{s}\right)\left(t-\tau \right)+\theta_{c}+\theta_{s}\right) \end{array}$$

Equation (12) is the basis for the subsequent design of a reception and processing algorithm. This representation of the B1-ADS signal can further reveal the similarity between itself and the regular BOC signal. Assuming that the baseband waveforms and the amplitudes of both the B1I and B1C signals are the same in Equation (12), the B1-ADS signal can be considered as a special BOC signal with a carrier frequency of 1568.259 MHz and a subcarrier frequency of 7.161 MHz. This explains the similarity of the ACFs of the B1-ADS and BOC(7,2) signals in Figure 3.

## 4. The ASYM-DBT Method and Thermal Noise Performance Analysis

Although the B1-ADS signal has characteristics similar to that of the BOC signal, it is very difficult to obtain and generate the true subcarrier waveform due to the two components of B1-ADS with unequal power and different modulation schemes. This renders those reception techniques of BOC signals that need to generate local subcarrier waveforms, such as Bump-Jump [15] and DET [16], impossible to apply to the B1-ADS signal. To tackle this problem, we start with the reception model given in Equation (12), extend the previous Dual BPSK Tracking method [17] designed for optimal reception of the AltBOC signal, and propose a novel Asymmetric Dual-Band Tracking technique to achieve the optimal reception processing of B1-ADS signal, which we abbreviate as ASYM-DBT.

### 4.1. The ASYM-DBT Method

**Figure 4:**
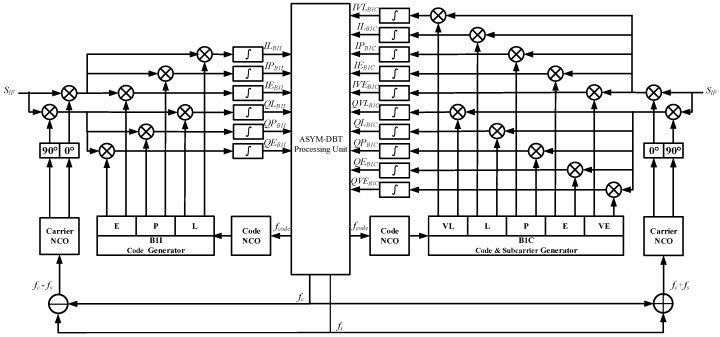
The tracking channel architecture of ASYM-DBT for the B1-ADS signal.

Figure 4 depicts the tracking channel architecture of ASYM-DBT for the B1-ADS signal, which consists of two groups of different types of correlators and one processing unit. Two groups of correlators on the left and right are used to correlate the received signal with locally generated B1I and B1C signals, respectively. Their outputs are then fed into the processing unit in the middle. The processing unit is responsible for combining the outputs of the two groups of correlators coherently or non-coherently, forming the discriminator outputs of the code, subcarrier, and carrier, and filtering them. Closed loops for code, subcarrier, and carrier tracking are finally formed by feeding their corrected NCO frequencies back to the correlators. Three kinds of measurements—code phase $\hat{\tau }$, subcarrier phase ${\hat{\theta }}_{s}$, and carrier phase ${\hat{\theta }}_{c}$—can be extracted from the corresponding loops. As can be seen in the next section, code tracking errors are on the order of decimeters, subcarrier tracking error is on the order of centimeters. Therefore, a natural idea is to combine the subcarrier phase measurement (which is high-precision but ambiguous) with the code measurement (which is just the opposite) to form a high-precision and unambiguous measurement given the subcarrier wavelength of 42.9 m, as shown below [17]:

(13) $${\hat{\tau }}_{c}={\hat{\theta }}_{s}+round(\frac{\hat{\tau }-{\hat{\theta }}_{s}}{\lambda })\times \lambda$$

where ${\hat{\tau }}_{c}$ represents the combined pseudorange measurement and λ represents the subcarrier wavelength.

The following describes the outputs of correlators and ASYM-DBT algorithms in the processing unit in detail.

#### 4.1.1. Correlator

In Figure 4 the local B1I signals generated on the left side and the local B1C signals generated on the right side are used to correlate with the incoming IF signal, respectively. The B1I correlator is the conventional BPSK-type correlator. The locally generated signals are defined as the six possible combinations of in-phase (I) and quadra-phase (Q) local carrier replicas and early (E), prompt (P), and late (L) local code replicas [1]. The B1C correlator is the classical BOC(1,1)-type correlator. The locally generated signals are defined as the ten possible combinations of in-phase and quadra-phase local carrier replicas and very early (VE), early, prompt, late, and very late (VL) local code replicas. This setting supports the Bump-Jump algorithm in detecting and recovering from a false lock in the single-band processing mode based on the comparison of the main and side peak amplitudes.

According to Equation (12), it is not difficult to discover that B1I and B1C signals are mutually orthogonal frequency-wise and code-wise. This means one signal has little influence on the correlator outputs of the other signal on the other sideband. For simplicity, and to clarify the concepts underlying the algorithm design, the thermal noise term in (2) is dropped. Thus, the outputs of the two groups of correlators can be approximately expressed as follows:

(14a) $$\left\{ \begin{array}{l} IE_{B1I}≃A_{B1I}D_{B1I}R_{B1I}(\Delta \tau -d_{B1I}/2)T\sin c(\alpha )\cos(\alpha +\Delta \theta_{c}-\Delta \theta_{s})\\ QE_{B1I}≃A_{B1I}D_{B1I}R_{B1I}(\Delta \tau -d_{B1I}/2)T\sin c(\alpha )\sin(\alpha +\Delta \theta_{c}-\Delta \theta_{s})\\ IP_{B1I}≃A_{B1I}D_{B1I}R_{B1I}(\Delta \tau )T\sin c(\alpha )\cos(\alpha +\Delta \theta_{c}-\Delta \theta_{s})\\ QP_{B1I}≃A_{B1I}D_{B1I}R_{B1I}(\Delta \tau )T\sin c(\alpha )\sin(\alpha +\Delta \theta_{c}-\Delta \theta_{s})\\ IL_{B1I}≃A_{B1I}D_{B1I}R_{B1I}(\Delta \tau +d_{B1I}/2)T\sin c(\alpha )\cos(\alpha +\Delta \theta_{c}-\Delta \theta_{s})\\ QL_{B1I}≃A_{B1I}D_{B1I}R_{B1I}(\Delta \tau +d_{B1I}/2)T\sin c(\alpha )\sin(\alpha +\Delta \theta_{c}-\Delta \theta_{s}) \end{array}\right.$$

(14b) $$\left\{ \begin{array}{l} IVE_{B1C}≃A_{B1C}R_{B1C}(\Delta \tau -d_{B1C})T\sin c(\beta )\cos(\beta +\Delta \theta_{c}+\Delta \theta_{s})\\ QVE_{B1C}≃A_{B1C}R_{B1C}(\Delta \tau -d_{B1C})T\sin c(\beta )\sin(\beta +\Delta \theta_{c}+\Delta \theta_{s})\\ IE_{B1C}≃A_{B1C}R_{B1C}(\Delta \tau -d_{B1C}/2)T\sin c(\beta )\cos(\beta +\Delta \theta_{c}+\Delta \theta_{s})\\ QE_{B1C}≃A_{B1C}R_{B1C}(\Delta \tau -d_{B1C}/2)T\sin c(\beta )\sin(\beta +\Delta \theta_{c}+\Delta \theta_{s})\\ IP_{B1C}≃A_{B1C}R_{B1C}(\Delta \tau )T\sin c(\beta )\cos(\beta +\Delta \theta_{c}+\Delta \theta_{s})\\ QP_{B1C}≃A_{B1C}R_{B1C}(\Delta \tau )T\sin c(\beta )\sin(\beta +\Delta \theta_{c}+\Delta \theta_{s})\\ IL_{B1C}≃A_{B1C}R_{B1C}(\Delta \tau +d_{B1C}/2)T\sin c(\beta )\cos(\beta +\Delta \theta_{c}+\Delta \theta_{s})\\ QL_{B1C}≃A_{B1C}R_{B1C}(\Delta \tau +d_{B1C}/2)T\sin c(\beta )\sin(\beta +\Delta \theta_{c}+\Delta \theta_{s})\\ IVL_{B1C}≃A_{B1C}R_{B1C}(\Delta \tau +d_{B1C})T\sin c(\beta )\cos(\beta +\Delta \theta_{c}+\Delta \theta_{s})\\ QVL_{B1C}≃A_{B1C}R_{B1C}(\Delta \tau +d_{B1C})T\sin c(\beta )\sin(\beta +\Delta \theta_{c}+\Delta \theta_{s}) \end{array}\right.$$

where $R_{B1I}\left(\Delta \tau \right)$ and $R_{B1C}\left(\Delta \tau \right)$ are the normalized ACFs of the B1I and B1C signals, respectively; $d_{B1I}$ and $d_{B1C}$ are the correlator spacing; T represents the pre-detection integration time; $\Delta \tau =\tau -\hat{\tau }$, $\Delta \theta_{s}=\theta_{s}-{\hat{\theta }}_{s}$, $\Delta \theta_{c}=\theta_{c}-{\hat{\theta }}_{c}$ are the residual errors for code, subcarrier, and carrier phases; and “^” denotes the previous estimates of the variable. Besides this, $\alpha =\pi (f_{c}-{\hat{f}}_{c}-f_{s}+{\hat{f}}_{s})T$ and $\beta =\pi (f_{c}-{\hat{f}}_{c}+f_{s}-{\hat{f}}_{s})T$ are introduced here to simplify the expressions. *D* represents the navigation message bit of the B1I signal because it does not have a pilot channel. Note that the amplitudes and ACFs of B1I and B1C are not the same due to the unequal power and different modulation schemes, and correlator spacing d, as an adjustable parameter, may be set to different values for two groups of correlators.

#### 4.1.2. Processing Unit

**Figure 5:**
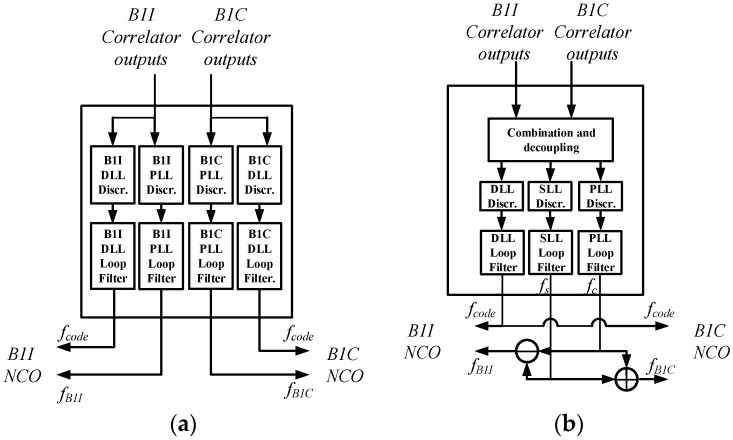
Two working modes of the processing unit. (**a**) Single-band mode; (**b**) Dual-band mode.

The processing unit supports two working modes of single-band and dual-band processing, and can smoothly switch between the two modes without any hardware modifications. While the single-band processing mode allows separate discriminating and filtering for the B1I and B1C signals at the same time, the dual-band processing mode is used to track the whole wideband signal, B1-ADS. Figure 5 depicts the processing flow of the two working modes. During the channel initialization phase, the single-band processing mode is first adopted to achieve reliable tracking of both the B1I and B1C signals, respectively and simultaneously. Once separate tracking obtains stable code and carrier parameters of both signals, the processing unit may shift smoothly to the dual-band processing mode to track the B1-ADS signal to obtain the optimal ranging performance. When one sideband undergoes narrow-band interference, the processing unit can shift back to the single-band mode to ensure that at least the signal on the other sideband can be reliably tracked.

Obviously, the dual-band working mode of the processing unit is more complicated and needs to deal with the asymmetry resulting from different modulation schemes and the unequal power of the upper and lower sidebands of B1-ADS. The following will detail those algorithms under this working mode.

• Discriminators for subcarrier and carrier phases

The phase discrimination results of both carrier and subcarrier are calculated based on the prompt branches of two groups of correlators. In the DBT method designed for the AltBOC signal, by virtue of the symmetry between the upper and lower bands and the existence of pilot channels, the coherent combinations of the correlator outputs are directly implemented with the aim of decoupling the subcarrier and carrier phases and hence obtaining their respective estimates. However, this method cannot be applied directly to the B1-ADS signal because the B1I and B1C signals have different power and the B1I signal is only a data channel modulated by the navigation message. Consequently, the carrier and subcarrier phase discriminations of the B1-ADS signal can only be acquired after stripping the B1I navigation message and compensating for the unequal power of the two signals.

To strip the navigation message of B1I, it is necessary to provide an estimation of the message data bit, ${\tilde{D}}_{B1I}$. This can be achieved in two ways [26]. When the signal-to-noise ratio meets the requirement of reliable demodulation, the estimation of the data bits of the message can be directly obtained from the sign of $IP_{B1I}$. Otherwise, the bits may be provided through the assistance data in an Assisted-BDS system.

To compensate for the unequal power of both signals, it is necessary to know the amplitude ratio between two signals, γ. Under the assumption that the local codes are approximately aligned with the received signal, for the prompt branches of two groups of correlators we have Δτ≈0; then $R_{B1C}(\Delta \tau )\approx 1$ and $R_{B1I}(\Delta \tau )\approx 1$. Because the propagation paths to the receiver antenna are the same, the amplitude ratio $A_{B1I}/A_{B1C}$ of the received signal is approximately equal to that of the transmitted signal from the satellite, i.e., $\gamma =2:\sqrt{3}$.

Given the estimated B1I data bit ${\tilde{D}}_{B1I}$ and the theoretical amplitude ratio of B1I and B1C γ, and further assuming that both the B1I and B1C are stably tracked—viz., α and β in Equation (14) approach zero—then the following coherent combinations can be formulated by applying the trigonometric function equation:

(15) $$\begin{array}{l} \gamma QP_{B1C}-{\tilde{D}}_{B1I}QP_{B1I}≃2A_{B1I}Tcos(\pi \Delta f_{c}T+\Delta \theta_{c})sin(\pi \Delta f_{s}T+\Delta \theta_{s})\\ \gamma QP_{B1C}+{\tilde{D}}_{B1I}QP_{B1I}≃2A_{B1I}T\sin(\pi \Delta f_{c}T+\Delta \theta_{c})\cos(\pi \Delta f_{s}T+\Delta \theta_{s})\\ \gamma IP_{B1C}+{\tilde{D}}_{B1I}IP_{B1I}≃2A_{B1I}T\cos(\pi \Delta f_{c}T+\Delta \theta_{c})\cos(\pi \Delta f_{s}T+\Delta \theta_{s}) \end{array}$$

As can be observed from Equation (15), subcarrier and carrier phases are completely separated. Then, phase discriminations of subcarrier and carrier can be achieved by employing the pure PLL discriminator by the optimal four-quadrant arctangent discriminator algorithm as follows:

(16) $$\begin{array}{ll} disc(\theta_{s}) & =\mathrm{atan}2(\gamma QP_{B1C}-{\tilde{D}}_{B1I}QP_{B1I},\gamma IP_{B1C}+{\tilde{D}}_{B1I}IP_{B1I})\\ & ≃\pi \Delta f_{s}T+\Delta \theta_{s}\\ disc(\theta_{c}) & =\mathrm{atan}2(\gamma QP_{B1C}+{\tilde{D}}_{B1I}QP_{B1I},\gamma IP_{B1C}+{\tilde{D}}_{B1I}IP_{B1I})\\ & ≃\pi \Delta f_{c}T+\Delta \theta_{c} \end{array}$$

Note that the pure PLL discriminator is superior to the Costas PLL discriminator with a 6 dB gain in signal tracking threshold [1].

• Discriminators for code phase

The coherent and non-coherent discrimination results of the code phase are calculated based on the early and late branches of two groups of correlators. In the design of the code phase discriminator for the B1-ADS signal, the difference in ACFs between the B1I and B1C signals and the difference of the correlator spacing between the two groups of correlators must be considered.

The coherent code phase discriminator of the B1-ADS signal is the weighted sum of the coherent discriminators of the B1I and B1C signals as follows [1,11]:

(17) $$disc_{Coh}(\tau )=\frac{1}{2}\frac{2-k_{B1I}d_{B1I}}{4k_{B1I}}\frac{{IE}_{B1I}-{IL}_{B1I}}{{IP}_{B1I}}+\frac{1}{2}\frac{2-k_{B1C}d_{B1C}}{4k_{B1C}}\frac{{IE}_{B1C}-{IL}_{B1C}}{{IP}_{B1C}}$$

where $k_{B1I}$ and $k_{B1C}$ represent the slopes of the main peaks of B1I ACF and B1C ACF, i.e., one and three chip^−1^, respectively.

Similarly, the non-coherent code discriminator of the B1-ADS signal is given in the form of the weighted sum of the non-coherent discriminators of the B1I and B1C signals, as written below:

(18) $$disc_{Non}(\tau )=\frac{1}{2}\frac{2-k_{B1I}d_{B1I}}{4k_{B1I}}\frac{IE_{B1I}^{2}+QE_{B1I}^{2}-IL_{B1I}^{2}-QL_{B1I}^{2}}{IE_{B1I}^{2}+QE_{B1I}^{2}+IL_{B1I}^{2}+QL_{B1I}^{2}}+\frac{1}{2}\frac{2-k_{B1C}d_{B1C}}{4k_{B1C}}\frac{IE_{B1C}^{2}+QE_{B1C}^{2}-IL_{B1C}^{2}-QL_{B1C}^{2}}{IE_{B1C}^{2}+QE_{B1C}^{2}+IL_{B1C}^{2}+QL_{B1C}^{2}}$$

• Loop filters

Since the outputs of the discriminators mentioned above are noisy, the discriminators of code, subcarrier, and carrier are followed by the corresponding loop filters to reduce the noise. In our design, two separate second-order filters are selected for DLL and SLL, while a third-order filter is selected for PLL. Note that carrier-aiding of the code phase and subcarrier phase might be employed in order to achieve more accurate measurements. The three filtered discriminator outputs are then used to drive code and carrier NCOs of the B1I and B1C signals, respectively. Since there is no essential difference between the DBT loop filters and ASYM-DBT loop filters, for simplicity this paper will not discuss different filter configurations; refer to [1,17] for details.

### 4.2. Theoretical Noise Analysis

After detailing the ASYM-DBT method, we analyze its theoretical ranging performance for the B1-ADS signals. For simplicity, thermal noise is generally treated as the dominant source of error for signal tracking. Generally, the thermal noise manifests in the code performance, the subcarrier performance, and the carrier performance.

According to Equation (12), we can safely ignore the interaction between adjacent frequency signals when analyzing the thermal noise performance. By first calculating the signal part and noise part of each single sideband, the combination correlations of the B1-ADS signal and theoretical errors induced by the thermal noise can then be obtained. For simplicity, this paper will not cover the derivation process. With the coherent DLL discriminator, the dual-band thermal noise can be obtained as follows [27,28]:

(19) $$\sigma_{tDLL}^{2}≃\frac{B_{nDLL}(\int_{-\beta_{r}/2}^{\beta_{r}/2}\left(w_{B1I}G_{B1I}(f)\sin^{2}(\pi fd_{B1I}T)+w_{B1C}G_{B1C}(f)\sin^{2}(\pi fd_{B1C}T))df\right)}{{\left(2\pi \right)}^{2}C/N_{0}{\left(\int_{-\beta_{r}/2}^{\beta_{r}/2}(w_{B1I}{fG}_{B1I}(f)\sin(\pi {fd}_{B1I}T)+w_{B1C}fG_{B1C}(f)\sin(\pi fd_{B1C}T))df\right)}^{2}},$$

where $B_{nDLL}$ represents the equivalent noise bandwidth of the code tracking loop; $w_{B1C}$ and $w_{B1I}$ are the power proportion of the B1C and B1I signals; G(f) represents the PSD of the single-sideband signal, which is normalized to unity over infinite bandwidth; $\beta_{r}$ represents the equivalent dual-band front-end bandwidth; $C/N_{0}$ represents the carrier-to-noise ratio of the total dual band; d represents the E-L correlator spacing. In case of a non-coherent discriminator, the joint dual-band thermal noise is shown below:

(20) $$\begin{array}{l} \sigma_{tDLL}^{2}≃\frac{B_{nDLL}(\int_{-\beta_{r}/2}^{\beta_{r}/2}\left(w_{B1I}G_{B1I}(f)\sin^{2}(\pi fd_{B1I}T)+w_{B1C}G_{B1C}(f)\sin^{2}(\pi fd_{B1C}T))df\right)}{{\left(2\pi \right)}^{2}C/N_{0}{\left(\int_{-\beta_{r}/2}^{\beta_{r}/2}(w_{B1I}{fG}_{B1I}(f)\sin(\pi {fd}_{B1I}T)+w_{B1C}fG_{B1C}(f)\sin(\pi fd_{B1C}T))df\right)}^{2}}\times \\ \left(1+\frac{B_{nDLL}(\int_{-\beta_{r}/2}^{\beta_{r}/2}\left(w_{B1I}G_{B1I}(f)\cos^{2}(\pi fd_{B1I}T)+w_{B1C}G_{B1C}(f)\cos^{2}(\pi fd_{B1C}T))df\right)}{TC/N_{0}{\left(\int_{-\beta_{r}/2}^{\beta_{r}/2}\left(w_{B1I}G_{B1I}(f)\cos(\pi fd_{B1I}T)df+w_{B1C}G_{B1C}(f)\cos(\pi fd_{B1C}T)df\right)\right)}^{2}}\right) \end{array}$$

The term in the second line represents the squaring loss.

Considering the asymmetric property of the B1-ADS, the associated subcarrier phase error is given by (21a) [27,28]:

(21a) $$\sigma_{tPLLS}^{2}≃\frac{B_{nPLLS}}{C/N_{0}\int_{-\beta_{r}/2}^{\beta_{r}/2}(w_{B1I}G_{B1I}(f)+w_{B1C}G_{B1C}(f))df}.$$

Similarly, the carrier phase error is given by (21b):

(21b) $$\sigma_{tPLLC}^{2}≃\frac{B_{nPLLC}}{C/N_{0}\int_{-\beta_{r}/2}^{\beta_{r}/2}(w_{B1I}G_{B1I}(f)+w_{B1C}G_{B1C}(f))df},$$

where $B_{nPLLS}$ and $B_{nPLLC}$ represent the equivalent noise bandwidth of the subcarrier and carrier tracking loops, respectively. These theoretical results of the thermal noise performance are compared with the experiment results in the next section.

## 5. Experiment and Discussion

In this section, a simulated experiment is carried out based on a software receiver and a GNSS signal simulator which supports the generation of the BDS global signals. In addition to demonstrating the ranging performance advantage of the B1-ADS signal over separate B1I or B1C signals, the experiment is also used to validate the feasibility of the ASYM-DBT method.

The software receiver is equipped with a wideband RF unit with a center frequency of 1570 MHz and a bandwidth of 52 MHz that can receive both B1I and B1C signals simultaneously. The entire B1 frequency band is converted to the intermediate frequency of 210 MHz in the RF unit, then is digitized with the sampling rate of 120 MHz, and fed into the PC for subsequent signal reception and processing. The main configuration of the PC platform is as follows: Intel(R) Core(TM) i7-6700K CPU, clocked 4.00 GHz, memory 16.0 GB, NVIDIA GeForce GTX TITAN X GPU. The software receiver can be configured as the following three receiving modes: processing the B1I signal independently, processing the B1C signal independently, and processing the B1-ADS signal with the ASYM-DBT algorithm. The pre-detection integration time T is set to 10 milliseconds, and the E-L correlation spacing of the B1I and B1C signals are set to one and one-half of the chip length, respectively. The noise bandwidth parameters of code, subcarrier, and carrier tracking loops are set to 0.4, 0.4, and 10 Hz, respectively.

In order to evaluate and compare the thermal noise performance of different receiving modes, a specific simulated signal scenario is set in the navigation signal simulator. Two different satellites (PRN 1 and PRN 2) are set to move with the same trajectory and broadcast both B1I and B1C signals simultaneously. Then, two independent channels are arranged in the receiver to process the signals of the two satellites, respectively. Therefore, the differences between measurement outputs of the two channels can be viewed as the thermal noise errors for code, subcarrier, and carrier tracking correspondingly. By adjusting the transmitted power, the standard deviation of the thermal noise errors can be obtained at different C/N_0_.

**Figure 6:**
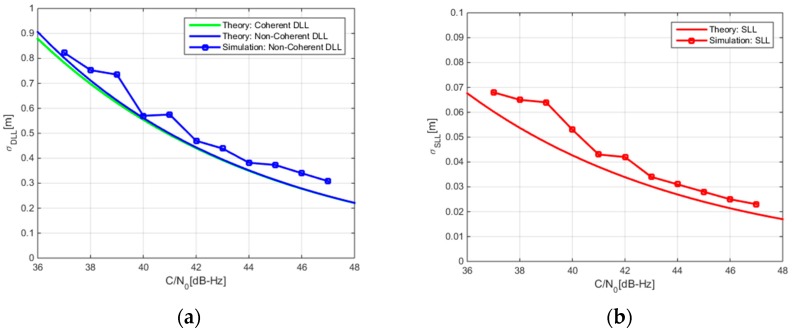

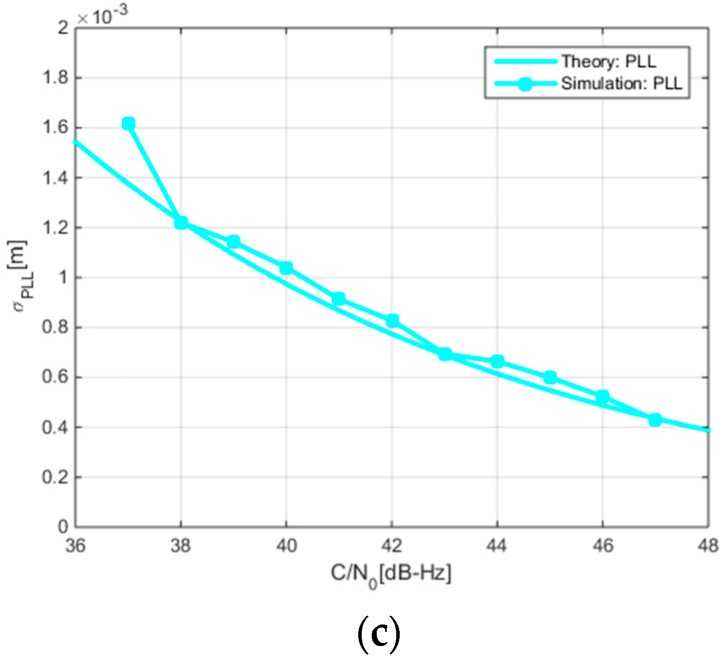
Comparison between the code, subcarrier, and carrier noise errors of B1-ADS. (**a**) Code noise error; (**b**) Subcarrier noise error; (**c**) Carrier noise error.

Figure 6 depicts the standard deviation of the code, subcarrier, and carrier tracking errors of the ASYM-DBT method while processing the B1-ADS signal. For the convenience of comparison, theoretically derived results are also presented in the same figure. Comparing SLL with DLL, the subcarrier noise error is one order of magnitude smaller than the code noise error. This is the result of the larger Gabor bandwidth of the B1-ADS signal. According to Equation (13), we can combine the subcarrier measurement with the code measurement to form a high-precision and unambiguous pseudorange measurement with centimeter-level accuracy. This high-precision pseudorange measurement is very helpful to rapidly determine the ambiguity of the carrier phase in Precise Point Positioning (PPP) or Real Time Kinematic (RTK) technology [29,30].

**Figure 7:**
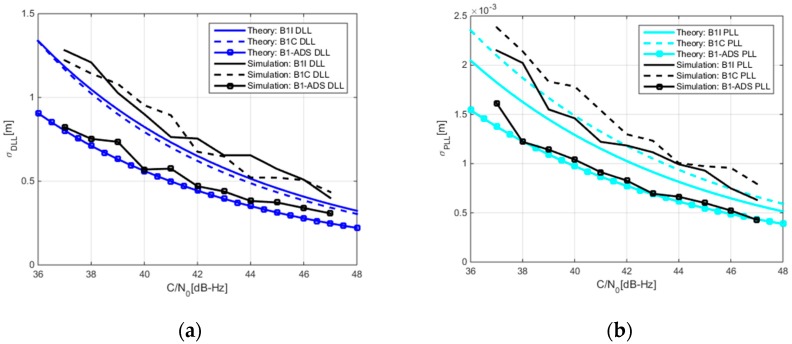
Comparison of code and carrier noise errors between B1I, B1C, and B1-ADS signals. (**a**) Code noise error; (**b**) Carrier noise error.

Figure 7 gives the thermal noise performance of DLL and PLL under three receiving modes. It must be noted that the horizontal axis in this figure is still expressed as C/N_0_ calculated based on the power of the B1-ADS signal. From Figure 7, it is obvious that the DLL noise performance of the B1-ADS signal is 3 dB better than that of B1I and B1C signals, and the PLL noise performance of the B1-ADS is also better than that of the other two signals. These results confirm that the ASYM-DBT method can effectively combine the B1I and B1C signals to obtain the power gain of the B1-ADS signal. It is worth mentioning that Figure 7a shows that the DLL noise performance of the B1I and B1C signals are almost the same. The reason is that the improved ranging performance brought by the steeper ACF of B1C signal is offset by the stronger power of the B1I signal relative to the B1C pilot signal.

## 6. Conclusions

This paper focuses on the optimal joint processing of B1I and B1C signals in the upcoming BeiDou global system. We propose to view these two signals as one navigation signal with asymmetric dual sidebands, named B1-ADS, whose Gabor bandwidth is much broader than that of B1I and B1C alone. To take full advantage of its broader Gabor bandwidth, the ASYM-DBT method has been designed to achieve optimal ranging accuracy with low hardware complexity. Theoretical analysis and experimental results based on the software receiver have proved the performance of the proposed method. Future research would extend the ASYM-DBT method to process other asymmetric dual sideband signals and to study the multipath mitigation algorithm under this method.

## Figures and Tables

**Table 1:** B1I and B1C Signal Structure.

Signal	Component	Center Frequency/MHz	Modulation	Phase/°	Power Proportion	Subcarrier/MHz	Pseudo-Random Code/Mcps
B1I	--	1561.098	BPSK(2)	0	1/2	--	2.046
B1C	pilot	1575.42	QMBOC ^1^	90/0	3/8	1.023/6.138	1.023
	data	1575.42	BOC(1,1)	0	1/8	1.023	1.023

^1^ Quadrature Multiplexed BOC.
